# Supplementary material for: Enhancement in acoustic focusing of micro and nanoparticles by thinning a microfluidic device
Source: R Soc Open Sci. 2019 Feb 20;6(2):181776. doi: 10.1098/rsos.181776 (PMC6408367; doi:10.1098/rsos.181776)
Supplement: Electronic Supplementary Material [file rsos181776supp1.docx]

**Enhancement in acoustic focusing of micro and nanoparticles by thinning a microfluidic device**

**Supplementary material**

Nobutoshi Ota^1^, Yaxiaer Yalikun^1,2^, Tomoyuki Suzuki^2^, Sang Wook Lee^3^, Yoichiroh Hosokawa^2^, Keisuke Goda^3,4^ and Yo Tanaka*^1^

^1^Center for Biosystems Dynamics Research (BDR), RIKEN, 1-3 Yamadaoka, Suita, Osaka 565-0871, Japan.

^２^Division of Materials Science, Nara Institute of Science and Technology, Ikoma, Takayama, Nara,630-0192, Japan

*^３^Department of Chemistry, School of Science, The University of Tokyo, 7-3-1 Hongo, Bunkyo-ku, Tokyo 113-0033, Japan*

*^4^ Japan Science and Technology Agency, 4-1-8, Honcho, Kawaguchi, Saitama 332-0012, Japan*

* E-mail: yo.tanaka@riken.jp


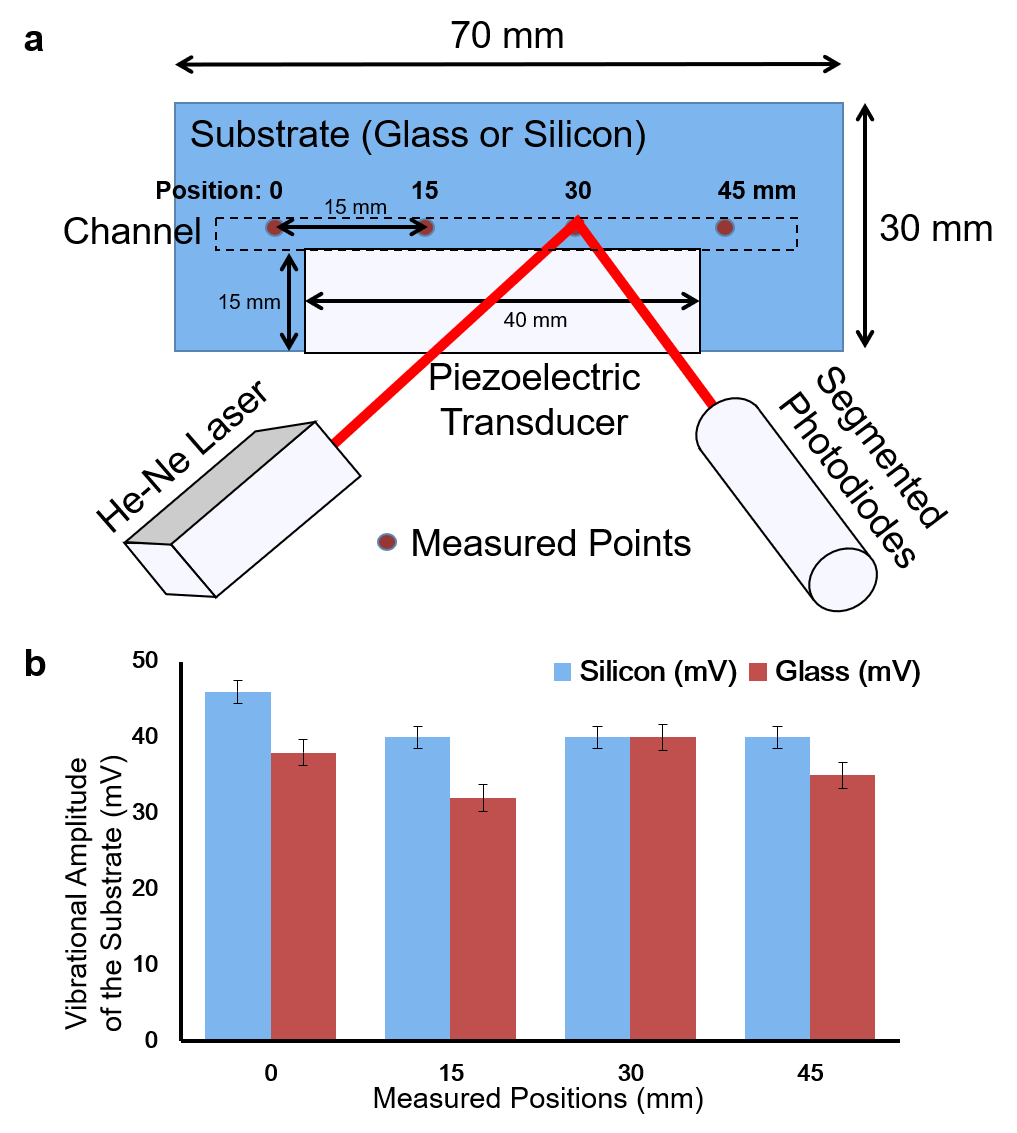


**Fig. S1** Measured amplitude of propagated acoustic waves on silicon and glass. (a) Illustration of the experimental setup to measure vibrational amplitude of propagated acoustic waves. Measured points were set on the area where a microchannel of 60-mm length was patterned on the etched plate. This area is indicated by dashed lines. (b) Summary of the measured vibrational amplitudes of acoustic waves on silicon and glass at the different points specified in (a).


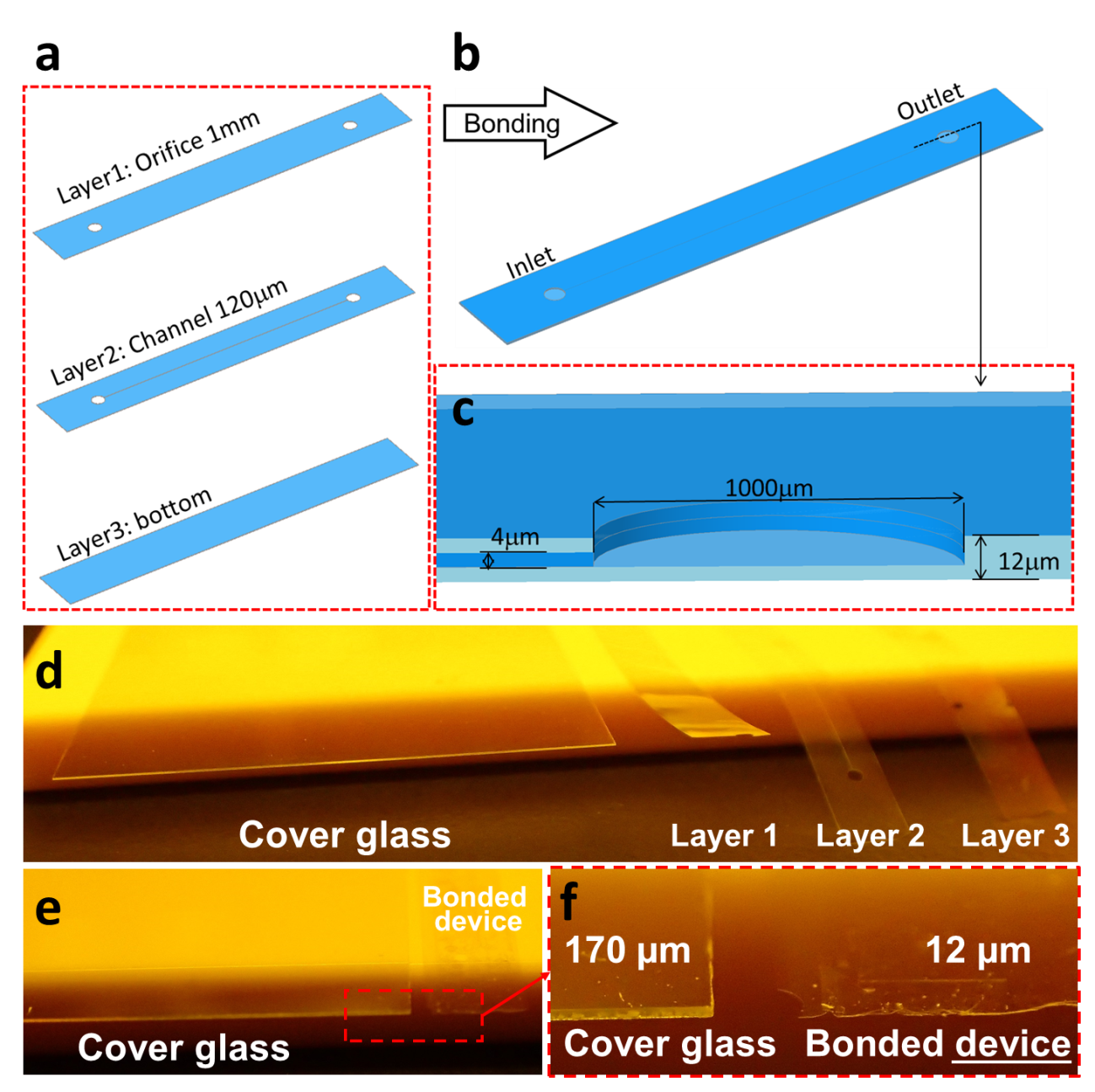


**Fig. S2** All-glass 12-μm thick ultrathin glass microfluidic device. (a) The ultrathin microfluidic device is composed of three glass layers of 0.004-mm thickness and with a microchannel of 4-μm height and 120-μm width. (b) Three layers were fusion-bonded together. (c) Inlet and outlet orifices are 1 mm in diameter. (d) Comparison of glass thickness: each layer of the glass microfluidic device and a cover glass (170-μm thickness). (e) Comparison between the thickness of the glass microfluidic device and a cover glass of 170-μm thickness. (f) Magnified view of the area enclosed by the red rectangle in (e). Scale bar is equal to 2 mm.


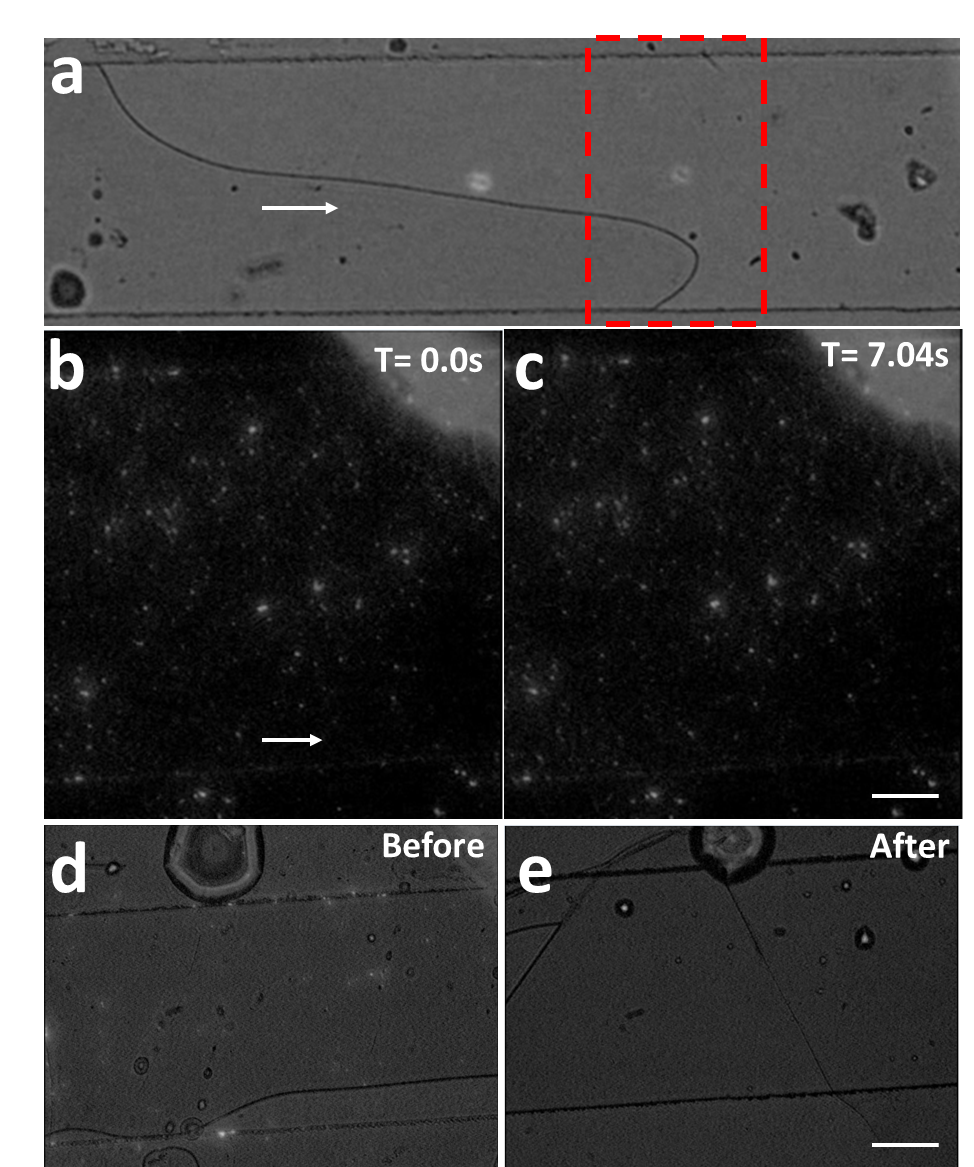


**Fig. S3** Acoustic waves applied to a 0.012-mm glass microfluidic device. (a) A functionality check was conducted by introducing air into the channel of the ultrathin glass microfluidic device in Fig. S1. The observation area is indicated by a red rectangle. (b) Dispersion of 280 nm beads when 0 V was applied to the piezoelectric transducer on the ultrathin device. (c) Dispersion of 280 nm beads when 20 V was applied. (d) The channel was undamaged before applying 20 V to the piezoelectric transducer. (e) By applying 20 V to the piezoelectric transducer, the generated acoustic waves cracked the ultrathin glass device. Introduced liquid leaked out from the device.


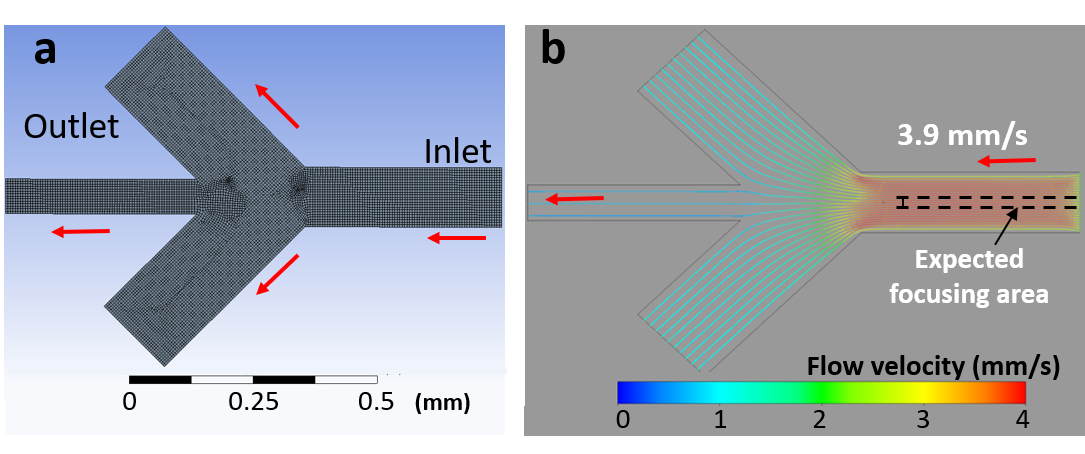


**Fig. S4** Simulation of the flow in a branched microchannel for enrichment of polystyrene beads. (a) A design of a branched microchannel for target collection was verified by using computational fluidic dynamics (CFD)-based simulation. Arrows indicate direction of liquid flow. (b) The targets in the expected focusing area were simulated as being collected in the middle (70-μm width) channel.


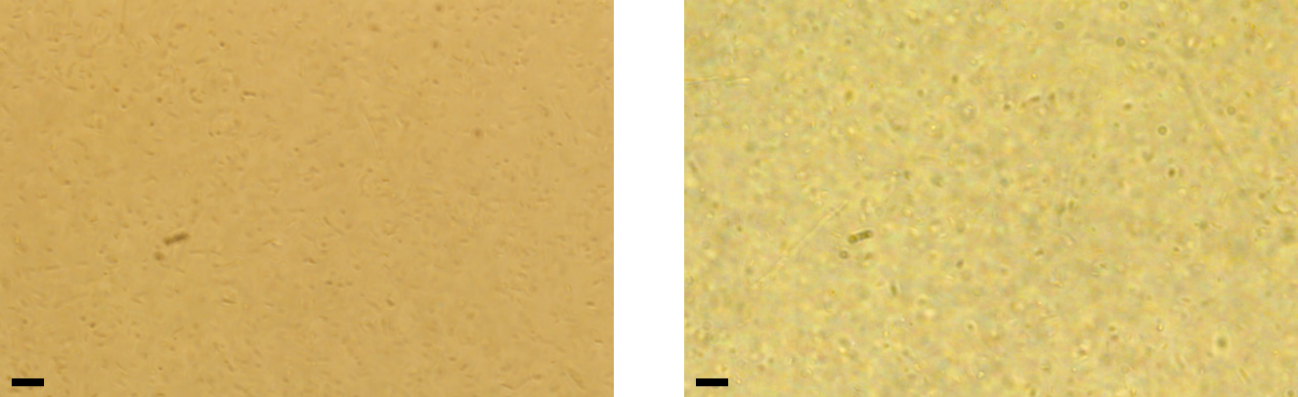


**Fig. S5** Verifying conditions of *E. coli*. *E. coli was* observed in a well (left) before and (right) after applying acoustic focusing in a microfluidic device. *E. coli* showed similar degree of activity in both conditions. Movie 3 (before acoustic focusing) and 4 (after acoustic focusing) are available. Scale bar: 20 μm.

**Movie 1** Enrichment of 10-μm beads with acoustic focusing (piezoelectric element actuated by 20 V) in a 0.4-mm glass microfluidic device. Scale bar: 50 μm.

**Movie 2** Enrichment of 10-μm beads with acoustic focusing (piezoelectric element actuated by 20 V) in a 1.8-mm glass microfluidic device. Scale bar: 50 μm.

**Movie 3** Conditions of *E. coli* before applying acoustic focusing. Scale bar: 20 μm.

**Movie 4** Conditions of *E. coli* after applying acoustic focusing. Scale bar: 20 μm.
